# The inhibitory effect of AMP-activated protein kinase (AMPK) on chemokine and prostaglandin production in human endometrial stromal cells

**DOI:** 10.1186/s12958-021-00867-1

**Published:** 2021-12-20

**Authors:** Yasushi Kawano, Hatsumi Sato, Kaori Goto, Masakazu Nishida, Kaei Nasu

**Affiliations:** Department of Obstetrics and Gynecology, Faculty of Medicine, Oita University, 1-1 Idaigaoka, Hasama, Yufu, Oita 879-5593 Japan

**Keywords:** Interleukin-1β, AMPK, Prostaglandin, Dysmenorrhea, Endometrial stromal cells

## Abstract

**Background:**

To investigate the role of adenosine monophosphate (AMP)-activated protein kinase (AMPK) on the production of interleukin (IL)-8, monocyte chemoattractant protein (MCP)-1, prostaglandin E2 and F2α induced by IL-1β in endometrial stromal cells (ESCs) following treatment with 5-aminoimidazole-4- carboxamide ribonucleoside (AICAR).

**Methods:**

Endometrial specimens were obtained and cultured. We examined the effects of IL-1β, IL-1 ra and AICAR on the production of IL-8, MCP-1, PGE2 and PGF2α in human ESCs. The phosphorylations of AMPK, IκB, 4EBP-1, p70S6K and S6 ribosomal protein were analyzed by Western immunoblotting.

**Results:**

Following stimulation by IL-1β, the production of IL-8, MCP-1, PGE2 and PGF2α showed significant increases, and these increases were suppressed by AICAR. The expression of cyclooxygenase-2 (COX-2) induced by IL-1β and suppressed by AICAR. The phosphorylation of IκB, 4EBP-1, p70S6K and S6 ribosomal protein were inhibited via an AMPK-dependent signal transduction.

**Conclusions:**

The production of IL-8, MCP-1, PGE2 and PGF2α induced by IL-1β in ESCs were involved in the negative regulatory mechanisms of AMPK. The substances that activate AMPK may be promising agents for the treatment of pathological problems such as dysmenorrhea.

## Introduction

Human endometrial stromal cells (ESCs) undergo morphologic and functional changes to provide the most suitable conditions for embryo implantation, which is regulated by several cytokines [1], polypeptide growth factors [2], steroid hormones, and eicosanoids including platelet-activating factor and prostaglandins (PGs) [3]. Cytokines are synthesized in the uterine endometrium, and ESCs were reported to produce and secrete a variety of cytokines including interleukin (IL)- 6, IL-8, macrophage colony-stimulating factor (MCSF) [4] and tumor necrosis factor (TNF)-α. PGs are one of the vasoactive substances thought to play a role in dilatation of microvessels and increased capillary permeability in the endometrium. PGs are released by both epithelial and stromal components of the endometrium and are thought to influence morphological and physiological changes in the endometrium. We demonstrated prostaglandin production by IL-1, and we found that this production was suppressed by an IL-1 receptor antagonist. Moreover, IL-1 also induced cyclooxygenase-2 (COX-2) in ESCs [3]. Dysmenorrhea induces painful uterine cramps during menstrual periods with no pathological evidence. It occurs in up to 50% of women with menstruation and can cause significant disruption in quality of life, and absentee [5]. It was demonstrated that an excessive amount of eicosanoids including PGs are released from the uterine endometrium during the menstrual period.

The adenosine monophosphate (AMP)-activated protein kinase (AMPK) is an energy-sensing enzyme with a heterotrimeric complex that is believed to be implicated in the regulation of energy metabolism at the intracellular and whole-organ levels [6–8]. It was reported that both energy-producing pathways and the down-regulation of energy-consuming processes are activated by AMPK [6, 7]. AMPK signaling of reproductive tissues in female is little known even if at the animal level. Decreasing steroidogenesis of ovarian granulosa cells by AMPK activation in animals has reportedly recognized [9]. The AMP analog, 5-aminoimidazole-4-carboxamide riboside (AICAR) is used as a pharmacological activator of AMPK. It is suspected that 5-aminoimidazole-4-carboxamide ribonucleoside (ZMP) is converted from AICAR by adenosine kinase in the cells. ZMP can activate AMPK according to its structural similarity with AMP, leading to AMPK activation [10].

Anti-inflammatory effect of AMPK was also implicated. It has been recognized that the AMPK activation plays an important role in inhibition of inflammatory response. It was reported that endotoxin lipopolysaccharide (LPS)-induced production of TNF-α, IL-1β, IL-6, inducible NOS (iNOS), and COX-2 were suppressed by AICAR in primary macrophages, microglia, astrocytes, and mesangial cells [10–13]. In addition, the constitutive COX-2 expression in colon cancer cells was suppressed by AMPK activation [14]. On the other hand, decreasing AMPK activity is associated with increasing of inflammation. The benefits of AMPK activation in research models of several inflammatory diseases were also demonstrated [15].

In the present study, we used ESCs as cell models to examine the effects of AMPK activation on the production of IL-1-mediated chemokines, or PGs via COX-2 gene expression. We postulated that the mechanism of anti-inflammatory reaction involving AMPK activation might mediate the direct effect of protein synthesis with inflammatory factors.

## Materials and methods

### Reagents

The following reagents were used: cell culture media RPMI 1640 (Nissui; Tokyo), fetal calf serum (FCS) (HyClone; Logan, UT), Hank’s balanced salt solution (HBSS; GIBCO-BRL, Gaithersburg, MD), collagenase (type I) and DNase (Sigma Chemical, St. Louis, MO), IL-1β and IL-1RA (R&D Systems, Minneapolis, MN).

AICAR and compound C were obtained from Sigma Chemical.

### Cell culture

Normal endometrial specimens were obtained from 10 premenopausal patients who had undergone hysterectomies for subserous myoma. All the specimens were classified as being in the late proliferative phase (days 9 to 12 of the menstrual cycle) on the basis of standard histologic criteria. This study was approved by the institutional review board of the Faculty of Medicine, Oita University, and written informed consent was obtained from all patients.

Normal ESCs were separated from epithelial glands by digestion of the tissue fragments with collagenase as described [1, 3] with a slight modification. Briefly, tissues were cut into 2- to 3-mm pieces and incubated with collagenase (200 IU/mL) and DNase (150 μg/mL) in HBSS with stirring for 2 h at 37 °C. The suspension was filtered through a 150-μm wire sieve to remove mucus and undigested tissues. The filtrate then was passed through a 80-μm wire sieve, which allowed the stromal cells to pass through while intact glands were retained. After being washed three times with serum-free RPMI 1640, the cells were transferred to culture flasks (Corning, New York, NY) at a density of 1 × 10^6^ cells/mL in RPMI 1640 supplemented with 10% heatinactivated FCS with penicillin (100 IU/mL) (GIBCO-BRL) and streptomycin (100 mg/mL) (GIBCO-BRL). The culture medium was replaced every 3 days. After two passages (10 to 12 days after isolation) by standard methods of trypsinization, the cells that had a purity of > 95% were used for the experiments. The cultures were incubated at 37 °C in 5% CO2 in air.

### Measurement of IL-8, MCP-1, PGE2 and PGF2α

For the evaluation of the productions of IL-8, MCP-1, PGE2 and PGF2α by ESCs, 5 × 10^5^ viable cells were plated on six-well culture plates (Corning) in 1 mL of culture medium with 10% FCS and cultured until they were fully confluent. The ESCs were washed twice with phosphate buffered saline (PBS) without calcium and magnesium, and were then added to serum-free medium. The supernatant was replaced with fresh culture medium containing various concentrations of IL-1 with AICAR for 24 h. Control cells received an equivalent volume of medium alone during the incubations.

AICAR is used as a pharmacological activator of AMPK. AICAR has cell permeability and can thus enter the ESCs. It is thought to be converted into 5-aminoimidazole-4-carboxamide ribonucleoside (AICA ribonucleoside, ZMP) by adenosine kinase. ZMP can activate the AMPK signaling pathway according to its structural similarity with AMP [16].

At the end of the culture period, the medium was stored at − 80 °C until assayed. These experiments were performed in triplicate and repeated four times. Commercially available enzyme-linked immunosorbent assays (ELISA) were used to determine the IL-8, MCP-1(R&D Systems) and PGE2 and PGF2α (Enzo Life Sciences, New York, NY) in the supernatants. The sensitivities of the assays for IL-8, MCP-1, PGE2 and PGF2α were 4.4 pg/mL, 5.0 pg/mL, 16.0 pg/mL and 5.0 pg/mL, respectively. The inter- and intra-assay coefficients of variance for the ELISAs were 9.6 and 7.8% for IL-8, 8.6 and 6.5% for MCP-1, 10.8 and 8.2% for PGE2, and 9.6 and 7.2% for PGF2α. These experiments were performed in triplicate and repeated three times.

### Protein preparation of ESCs and Western immunoblotting analysis (ECL-WB)

To investigate the intracellular signal transduction system in the ESCs, we performed a Western immunoblotting analysis as described (1). Briefly, 1 × 10^6^ cells were plated on a 100 mm dish (Nalgene Nunc, Rochester NY) in 10 mL of culture medium with 10% FCS and cultured until they were fully confluent. The supernatant was replaced with fresh culture medium containing IL-1 and AICAR. At the end of the culture period, the ESCs were washed twice with cold PBS without calcium or magnesium, harvested, pelleted, and lysed in ice-cold buffer containing 10 mM HEPES (pH 7.9), 10 mM KCl, 0.1 mM ethylenediaminetetraacetic acid (EDTA; pH 8.0), 0.1 mM ethylene glycol tetraacetic acid (EGTA), 1 mM dithiothreitol (DTT), 0.5 mM phenylmethanesulfonyl fluoride (PMSF), and 0.3 μg/mL leupeptin.

The cell lysate was centrifuged for 10 min at 3000×g in order to pellet the nuclei. The supernatant fractions were collected and centrifuged for 10 min at 10,000×g. The protein content was determined using a microbicinchoninic acid assay (Pierce, Rockford, IL) with bovine serum albumin (BSA) as a standard. The lysate was mixed with loading buffer (200 mM Tris-HCl [pH 7.9], 7% sodium dodecyl sulfate [SDS; w/v], 30% glycerol [v/v], 15% 2-mercaptoethanol [v/v], 0.75% bromophenol blue [w/v] and heated at 95 °C for 10 min. In each sample, 10 μg of protein was applied per lane. The blotted membranes were blocked in PBS containing 5% skim milk (Difco, Detroit, MI) for 1 h at room temperature and washed with three changes of Tris-buffered saline (TBS; 20 mM Tris, 137 mM NaCl, pH 7.6) buffer containing 0.1% Tween 20 for 15 min at room temperature.

The blotted membranes were then incubated and reacted overnight with 1:1000-diluted primary antibody (human phospho-AMPK antibody and AMPK antibody, rabbit polyclonal immunoglobulin G [IgG; Cell Signaling Technology, Beverly, MA], human COX-2 antibody [IgG, R&D Systems, MN], human IκB-α [IgG; Cell Signaling Technology], human 4EBP-1 [IgG; Cell Signaling Technology], human p70S6 kinase [IgG; Cell Signaling Technology], human S6 ribosomal protein [IgG; Cell Signaling Technology] and human glyceraldehyde-3-phosphatedehydrogenase [GAPDH] antibody, [IgG, Ambion Austin, TX] in TBS containing 5% BSA at 4 °C.

After being washed with three changes of TBS containing 0.1% Tween 20, the blotted membranes were incubated and reacted with 1:2000-diluted peroxidase-conjugated secondary antibody (anti-rabbit immunoglobulin γ or μ chain; Jackson Immunoresearch Laboratories, West Grove, PA) in TBS containing 5% BSA for 1 h at room temperature. After the membranes were washed with four changes of TBS containing 0.1% Tween 20, Lumi GLO from a Phototope-HRP Western Detection Kit (GE Healthcare UK, Buckinghamshire, England) was added to the blotted membranes and reacted for 1 min. The membranes were then covered with plastic wrap and exposed to X-ray film (GE Healthcare UK) for 1–2 min.

## Statistical analysis

The data are presented as mean ± standard deviation (SD) and were analyzed using the Bonferroni-Dunn test. *P-values* < 0.05 were considered significant. The confidence intervals with *p*-values for multiple statistical analyses are at the 95% level.

## Results

### AMPK phosphorylation was induced by AICAR

**Fig. 1:**
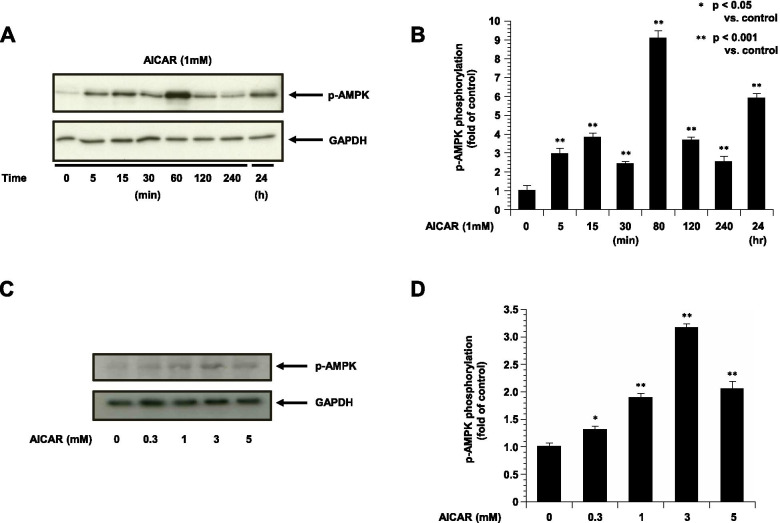
The phosphorylation of pAMPK in ESCs treated with AICAR (3 mM) for 0 min to 24 h (*n* = 4). GAPDH is shown as the internal control. **A**: Representative blots illustrating the effect of AICAR treatment on pAMPK and GAPDH for 0 min to 24 h. **C**: Representative blots illustrating the effect of AICAR (0–5 mM) treatment on pAMPK and GAPDH for 30 min. The blot layout is in the same order as the graph in panels **B** and **D**, respectively. *P < 0.05 vs. controls. **P < 0.001 vs. controls

To elucidate the role of AMPK in the ESCs, we examined the effects of AICAR on the phosphorylation of AMPK. We found that 1 mM AICAR activated AMPK time-dependently compared to the controls (Fig. 1A, B). Treatment with AICAR induced a greater amount of AMPK phosphorylation in 1 to 3 mM in dose-dependently increased (Fig. 1C, D). We evaluated the activity of AMPK by monitoring the phosphorylation of AMPK on Thr172, which is required for the activation of AMPK [17].

### AICAR inhibited the production of COX-2 in ESCs following stimulation by IL-1β

**Fig. 2:**
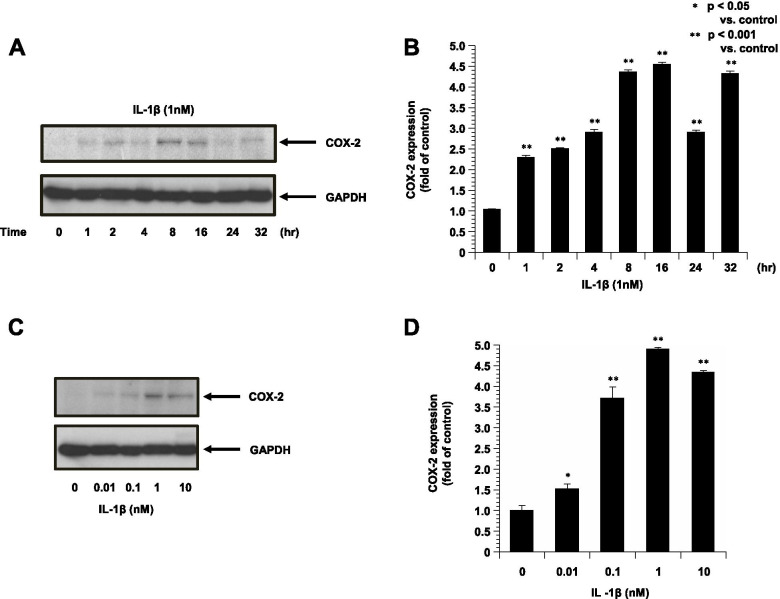
The expression of COX-2 in ESCs treated with IL-1β is shown (*n* = 4). GAPDH is shown as the internal control. **A**: Representative blots illustrating the effect of IL-1β (1 nM) treatment on COX-2 and GAPDH for 0 min to 32 h. **C**: Representative blots illustrating the effect of 0–1- nM IL-1β treatment on COX-2 and GAPDH for 16 h. The blot layout is in the same order as the graph in panels **B** and **D**, respectively. *P < 0.05 vs. controls. **P < 0.001 vs. controls

The results of the Western blot analysis demonstrated that COX-2 expression in the ESCs was stimulated by IL-1β. The expected 75-kDa band corresponding to COX-2 was found. Treatment with IL-1β induced a greater cellular protein levels of COX-2 in 8–16 h (Fig. 2A, B) and the dose-dependently increased (Fig. 2C, D).

**Fig. 3:**
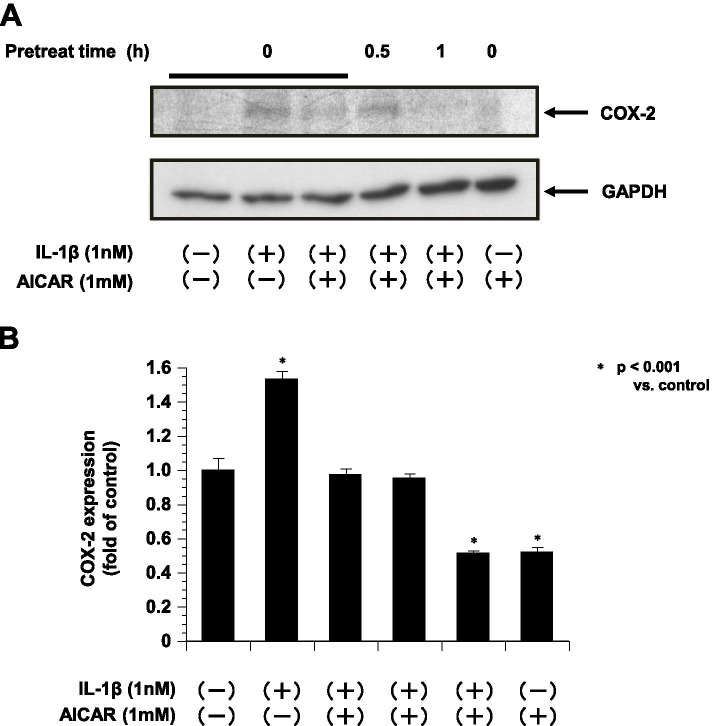
The expression of COX-2 in ESCs treated with IL-1β and AICAR is shown (*n* = 4). GAPDH is shown as the internal control. **A**: Representative blots illustrating the effect of IL-1β (1 nM) treatment on COX-2 and GAPDH by and/or pretreatment with AICAR (1 mM) for 16 h. The blot layout is in the same order as the graph in panel **B**. *P < 0.001 vs. controls

**Fig. 4:**
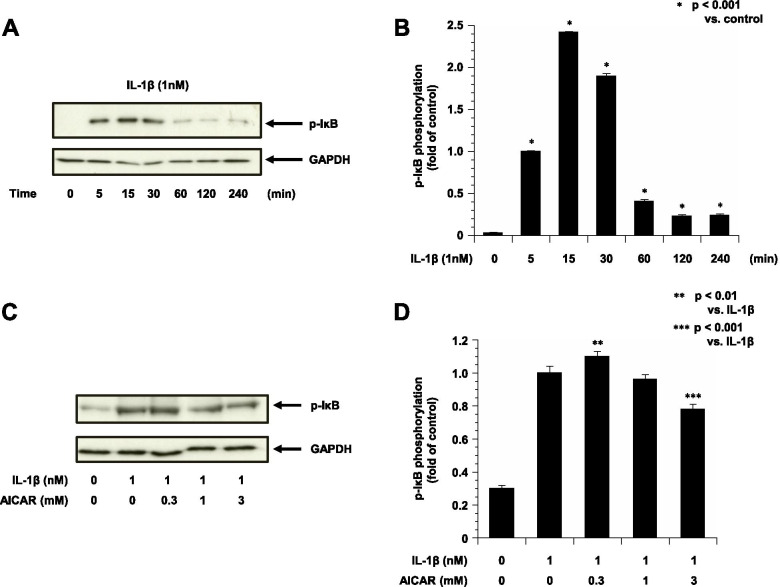
The phosphorylation of IκB in ESCs treated with IL-1β with or without AICAR (*n* = 4). GAPDH is shown as the internal control. **A**: Representative blots illustrating the effect of IL-1β (1 nM) treatment on IκB and GAPDH for 0–240 min. The blot layout is in the same order as the graph in panels **B**. **C**: Representative blots illustrating the effect of IL-1β (1 nM) treatment and/or pretreatment with AICAR (0.3-3 mM) for 15 min on IκB and GAPDH. The blot layout is in the same order as the graph in panel **D**. **P < 0.01 vs. IL-1β only. ***P < 0.001 vs. IL-1β only

The anti-inflammatory effect of AICAR was also apparent when the cells were pretreated with AICAR for only 1 h and then stimulated for 16 h with IL-1β. As shown in Fig. 4, treatment with the combination of IL-1β and AICAR resulted in reductions in the cellular protein levels of COX-2 compared to treatment with IL-1β alone (Fig. 3A, B).

### AICAR inhibited IκB phosphorylation in ESCs following stimulation by IL-1β

To elucidate the mechanism of the IL-1β-induced secretions of IL-8, MCP-1, PGE2 and PGF2α by ESCs, the effects of IL-1β-specific mechanism, the phosphorylation of IκB, were examined.

The results of the Western blot analysis demonstrated that the IκB phosphorylation in the ESCs was stimulated by IL-1β. The expected 37-kDa band corresponding to IκB was found (Fig. 4). Treatment with IL-1β induced greater cellular protein levels of IκB at 5–30 min (Fig. 4A, B). As shown in Fig. 4C, D, treatment with the combination of IL-1β (1 nM) and AICAR (3 mM) resulted significantly in reductions in cellular protein levels of IκB phosphorylation compared to treatment with IL-1β alone. However, IL-1β (1 nM) and AICAR (0.3 mM) resulted in rising in cellular protein levels of IκB phosphorylation.

### AICAR activated AMPK and inhibited translation-related proteins in the ESCs

We examined the effects of AICAR on AMPK in ESCs. We evaluated the activity of AMPK by monitoring the phosphorylation of AMPK on Thr172, which is required for the activation of AMPK. We next examined the effects of AICAR on mTORC1 signaling, which is negatively regulated by AMPK and a major regulator of translation initiation. We assessed the phosphorylation status of the two direct downstream targets, 40S ribosomal S6 kinase (p70S6K) (T389) and the eukaryotic translation initiation factor 4E (eIF4E)-binding protein 1 (4E-BP1) (T37/46).

**Fig. 5:**
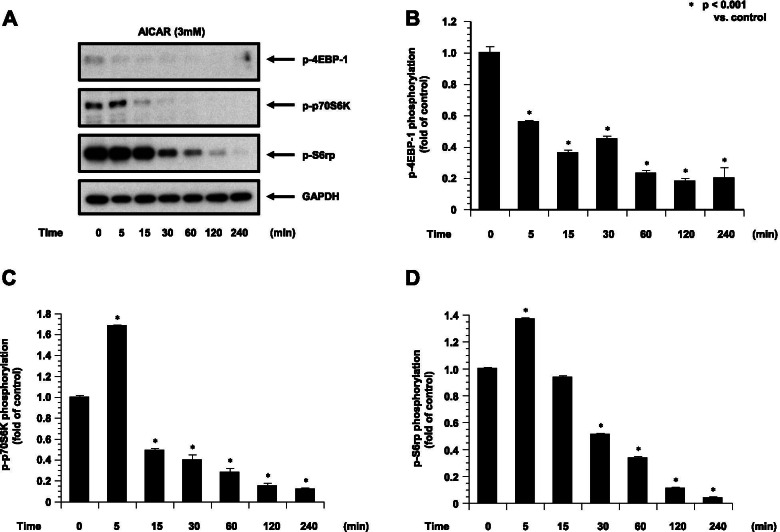
The phosphorylation of p4E-BP1, p70S6kinase and S6 ribosomal protein in ESCs treated with AICAR (3 mM) is shown (*n* = 4). The phosphorylation of p4E-BP1, p70S6K and S6 ribosomal protein was quantified against GAPDH. **A**: Representative blots illustrating the effect of treatment on p4EBP-1 and GAPDH by AICAR (3 mM) for 0–240 min (*n* = 4). The blot layout is in the same order as the graph in panels **B** (4EBP-1), **C** (p70S6K), **D** (S6 ribosomal protein), respectively. *P < 0.001 vs. controls

**Fig. 6:**
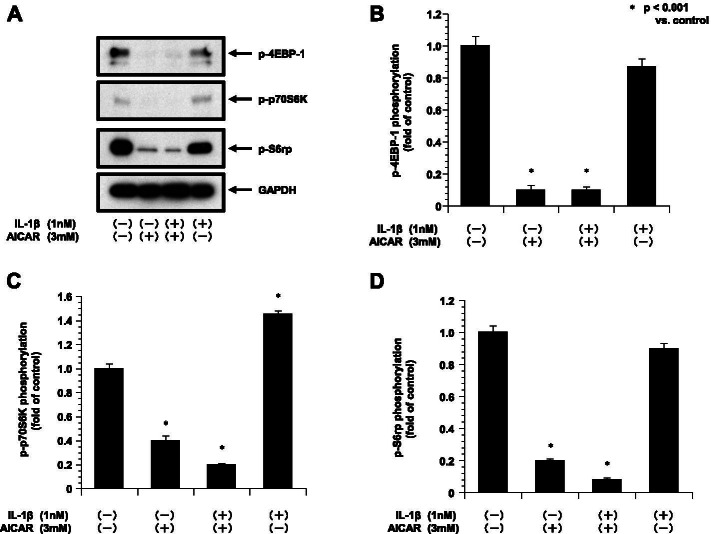
The phosphorylation of p4EBP1, p70S6kinase and S6 ribosomal protein in ESCs treated with IL-1β with or without AICAR is shown (*n* = 4). The phosphorylation of p4E-BP1, p70S6K and S6 ribosomal protein was quantified against GAPDH. **A**: Representative blots illustrating the effect of treatment on p4EBP-1 and GAPDH by IL-1β (1 nM) with or without AICAR (3 mM) for 240 min (*n* = 4). The blot layout is in the same order as the graph in panels **B** (4EBP-1), **C** (p70S6K), and **D** (S6 ribosomal protein), respectively. *P < 0.001 vs. controls

We found that AICAR time-dependently decreased the phosphorylation of 4E-BP1. AICAR treatment also decreased the p70S6K (T389) phosphorylation, resulting in S6 ribosomal protein (Fig. 5). This inhibition was also detected by the treatment with AICAR and IL-1β. The phosphorylation of 4E-BP1, p70S6K and S6 ribosomal protein were reduced by AICAR and IL-1β treatment (Fig. 6).

### AMPK inhibitor recovered the production of IL-8, MCP-1, PGE2 and PGF2α in ESCs following stimulation by IL-1 and AICAR

**Fig. 7:**
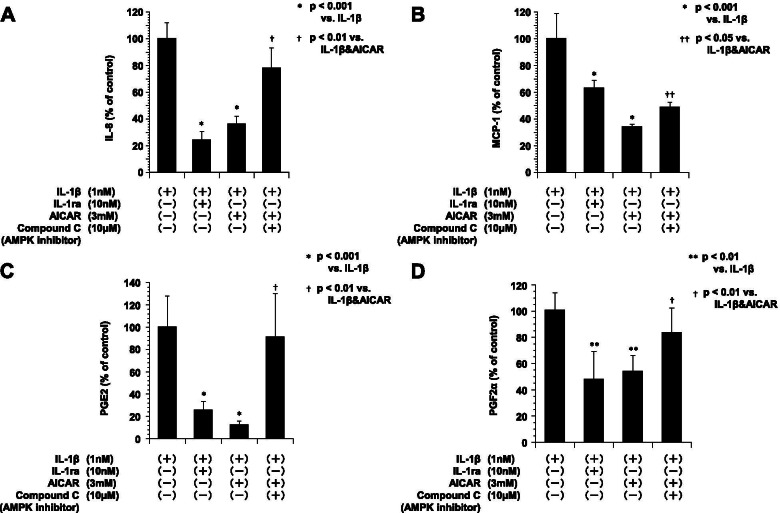
The levels of IL-8 (**A**), MCP-1 (**B**), PGE2 (**C**) and PGF2α (**D**) in culture media of ESCs after 24 h of stimulation with IL-1β, IL-1 receptor antagonist, AICAR and AMPK inhibitor (compound C). The ESCs were treated with human IL-1β (1 nM), IL-1ra (10 nM), AICAR (3 mM) and compound C (10 μM). *P < 0.001 vs. IL-1β (1 nM) stimulation (Bonferroni-Dunn test). The data are expressed as mean ± SD of triplicate samples from four separate representative experiments. **A**: *p < 0.001, (IL-1β vs. IL-1β + IL-1ra and IL-1β + AICAR), **p < 0.01 (IL-1β + AICAR vs. IL-1β + compound C). **B**: *p < 0.001, (IL-1β vs. IL-1β + IL-1ra and IL-1β + AICAR), **p < 0.05 (IL-1β + AICAR vs. IL-1β + compound C). **C**: *p < 0.001, (IL-1β vs. IL-1β + IL-1ra and IL-1β + AICAR), **p < 0.05 (IL-1β + AICAR vs. IL-1β + compound C). **D**: *p < 0.001, (IL-1β vs. IL-1β + IL-1ra and IL-1β + AICAR), **p < 0.05 (IL-1β + AICAR vs. IL-1β + compound C)

We examined the effect of AMPK inhibitor in IL-1β and AICAR treatment by ESCs for 24 h. As illustrated in Fig. 7, IL-1β caused a significant increase in IL-8, MCP-1, PGE2 and PGF2α release. When the ESCs were treated with IL-1β plus IL-1 ra or AICAR, the levels of IL-8, MCP-1, PGE2 and PGF2α were significantly decreased as compared to IL-1β alone. However, Compound C, an AMPK inhibitor, recovered the levels of IL-8, MCP-1, PGE2 and PGF2α suppressed by AICAR.

Cell counts at 24 h were substantially the same whether or not the cells were treated with IL-1β. During this period, the concentrations of IL-8, MCP-1, PGE2 and PGF2α in the media without cells were significantly lower (data not shown).

## Discussion

Cytokines, chemokines or PGs production in the endometrium might play pivotal roles in the mechanism of menstruation in humans. However, the exact mechanism or modulation in the endometrium has not been clarified in detail. It was reported that a large number of macrophages are distributed in human endometrium, and the presence of IL-1 proteins and mRNA in both stromal and epithelial cells of human endometrium has also been reported [18].

The endometrium has been demonstrated to express IL-1α, IL-1β, IL-1 receptor, and IL-1RA throughout the menstrual cycle [18]. Potentially an essential mediator in local intracellular interactions in endometrial tissues, IL-1 has also been known to be a potent stimulus in regulating the production of cytokines.

The result of the present study, demonstrated that AMPK activated by AICAR exerts its action on IL-1-induced IL-8, MCP-1PGE2 and PGF2α production in ESCs. In addition, IL-1-induced COX-2 protein expression levels were accordingly abolished by AICAR in these cells. It was shown that AICAR inhibits TNF-α and IL-1β-induced nuclear factor (NF)-κB reporter gene expression dose-dependently in immune cells [11, 12, 19, 20] and iNOS and COX-2 expression in stimulated macrophages [10]. It was also reported that activation of AMPK diminished the secretion of IL-8 by LPS in bovine endometrium [21]. Therefore, it is likely that AMPK acts to limit inflammatory reaction. According to the anti-inflammatory effects of AICAR, we hypothesized that AMPK activated by AICAR may decrease the production of inflammatory mediators in ESCs by attenuating the IL-1-mediated signal transduction cascade.

It has been demonstrated that AICAR inhibits the expression of IL-8 and COX-2 by inhibiting the phosphorylation and degradation of IκB-α [22]. Modulation of NF-κB was observed by activation of AMPK [23]. Our data suggest that AICAR might suppress IL-1-induced NF-κB activation before blocking IκB phosphorylation. Thus, our findings further demonstrated the modulation of IL-1-stimulated IκB-α phosphorylation by AMPK. It has been reported that AICAR attenuates the lipopolysaccharide-induced activation of NF-κB via the down-regulation of IκB kinaseα/β activity in glial cells [11]. This is the same mechanism that we observed in ESCs, suggesting that AMPK activation may inhibit cytokine-induced NF-κB activation by suppressing IκB activity. Moreover, PGE2 production is reduced by AMPK activation [20].

Our findings demonstrated that AICAR inhibited mTORC1 signaling, as manifested by the dephosphorylation of S6K1, 4E-BP1, and S6 in ESC. It has been investigated that a shift from the hyperphosphorylated form to the hypophosphorylated form of the phosphorylation state of 4E-BP1 was also caused by metformin [24]. It was also reported that the hypophosphorylated form of 4E-BP1 repressed the initiation of translation by binding to eIF4E with high affinity. It was also reported that activated AMPK reduced the activity of mTOR by phosphorylating TSC2, resulting to inhibit the mTOR by activation of TSC1-TSC2 complex. AMPK activated by AICAR has also been recognized to reduce the activity of p70S6K [25]. The importance of AICAR-induced inhibition of translation signaling and its relationship to the cellular functional regulation in ESCs should be investigated further.

PGs were indicated to be elevated in menstrual extracts of women who suffered primary dysmenorrhea compared to eumenorrheic women. Indeed, the increasing secretion of endometrial PGF2α during the menstrual phase was postulated in most but not all women with primary dysmenorrhea [5]. As the release of PGs into the menstrual fluid is a continuous or discontinuous process, the accumulation of PGs varies during menstruation [5]. The efficacy of cyclooxygenase inhibitors and estrogen-progestin oral contraceptive pills (OCPs) is supported by evidence-based data. Cyclooxygenase inhibitors have been recognized to reduce the amount of menstrual prostanoids released, with a concomitant reduction in uterine hypercontractility. OCPs inhibit endometrial development and decrease menstrual prostanoids, but OCP use also has a risk of deep venous thromboembolism. The activation of AMPK may contribute to suppress the production of prostanoids without any side effect. Apart from that, endometriosis occurs proinflammatory microenviroment in the early stages and to the pro-fibrotic activity of the advanced stages [26]. In addition, bone marrow-derived stem cells (BMDSCs) may migrate through peripheral circulation and cause endometriosis [27]. Our result may contribute to control the related substances such as chemokine.

A limitation of our study is that we showed the protein synthesis of only chemokines and COX-2 in ESCs. Further examination will be necessary to elucidate this modulation by AICAR via AMPK in other inflammatory substances. Our present findings indicate that AMPK activation may help reduce dysmenorrhea, which is believed to be increased by prostaglandins or substances from the inflammatory cells in menstrual fluid.

In conclusion, we found that through AMPK activation, AICAR inhibits the IL-1-stimulated IκB/ NF-κB-mediated pathway. These findings indicate the signal transduction pathways regulated by AMPK activation and suggest that AMPK activation can inhibit the production of inflammatory mediators in ESCs, resulting in the attenuation of dysmenorrhea. Further studies are needed to clarify the suppressive mechanism of AMPK signaling that could be beneficial in the treatment of particular human pathologies such as dysmenorrhea.

## Data Availability

Literature search results are available from the authors on reasonable request.

## Abbreviations

AICAR: 5-amino-imidazole-4-carboxyamide-1- β-D-ribofuranoside
AMPK: Adenosine monophosphate-activated protein kinase
COX: Cyclooxygenase
DTT: Dithiothreitol
EDTA: Ethylenediaminetetraacetic acid
EGTA: Ethylene glycol tetraacetic acid
GROα: Growth-regulated oncogene α
IL-6: Interleukin-6
IL-8: Interleukin-8
NFκB: Nuclear factor-κB
PG: Prostaglandin
PMSF: Phenylmethanesulfonyl fluoride
TNF-α: Tumor necrosis factor-α
